# A scoping review on the application of intersectionality as an analytical approach in primary care research

**DOI:** 10.1186/s12913-026-14873-9

**Published:** 2026-08-03

**Authors:** Saad Abbas, Harriet Bullen, Philip Britteon, Stephanie Gillibrand, Georgia Chatzi, Pulkit Singh, Sean Urwin

**Affiliations:** 1 Health, Organisation, Policy and Economics (HOPE) Group, School of Health Sciences, University of Manchester, Manchester, UK; 2 Cathie Marsh Institute for Social Research, School of Social Sciences, University of Manchester, Manchester, UK

**Keywords:** Intersectionality, Primary care, Healthcare, Inequality, Inequity, Disparity, Intercategorical, Intracategorical

## Abstract

**Background:**

While inequalities in primary care are well-documented, far less attention has been paid to how these vary when social identities, geographic contexts, and economic disadvantage combine. Intersectionality theory provides a critical framework for understanding how overlapping social identities can jointly shape individuals’ experiences. Despite its potential, little is known about how this theory has been applied to investigate inequalities in primary care, including the methods used and the populations or outcomes examined.

**Aim:**

We aim to map how intersectionality theory and methodologies have been applied to investigate inequalities in primary care.

**Method:**

We conducted this scoping review following PRISMA-ScR guidelines. We searched six databases and grey literature sources (up to June 2023) for empirical studies applying intersectionality in primary care contexts. Data was extracted and synthesised descriptively, categorising studies by their research methodology and intersectional approach.

**Results:**

Out of 1612 retrieved records, 37 studies met the inclusion criteria, with 86.5% (*k* = 32) published after 2017. Included studies were mainly based in high-income countries. Quantitative studies (*k* = 20) outnumbered qualitative (*k* = 15), highlighting a reliance on additive statistical models rather than experiential depth. Race and gender were the most frequently examined intersections. Studies primarily focused on patient outcomes, largely exploring disadvantage, with limited attention to provider perspectives or focused on the evaluation of interventions. Methodologically, more studies compared social groups (intercategorical) than explored unique subgroup experiences (intracategorical), and the application of intersectionality often served more as a guiding lens than a rigorous analytical tool.

**Conclusion:**

Intersectionality as an applied analytical approach in primary care research is relatively new but growing. It offers significant potential to reveal complex inequalities missed by more commonly applied descriptive or regression approaches. Key gaps remain regarding the geographic and demographic scope of research, the range of quantitative methods used, and the application of intersectional findings to develop and test interventions. Explicitly adopting intersectional approaches to identify those facing the greatest barriers in care is crucial to allow primary care to meet population needs.

**Clinical trial number:**

Not applicable.

**Trial registration:**

This scoping review adhered to a protocol previously published in BMC Systematic Reviews, and that is registered on the Open Science Framework website (https://osf.io/h9p83).

**Supplementary Information:**

The online version contains supplementary material available at https://doi.org/10.1186/s12913-026-14873-9.

## Introduction

Inequalities in health and care outcomes have long been a global concern. They became increasingly visible during the COVID-19 pandemic, which disproportionately affected socioeconomically disadvantaged groups by limiting access to essential services and medication [1, 2]. While there is a substantial body of evidence on primary care inequalities, highlighting disparities across socioeconomic status, ethnicity, gender, and other social determinants [3], far less is known about how these disadvantages interact. Much of the existing literature on inequalities in primary care access and experience [4–6] has examined single factors in isolation [7–10] or considered multiple factors independently without exploring their combined effects [11, 12]. This risks obscuring the compounded challenges faced by individuals at the intersection of multiple disadvantages and may lead to misidentification of the most vulnerable groups in society when social, economic, and geographical factors interact to produce specific disadvantages and experiences of discrimination [13]. This dynamic is explained through the concept of intersectionality.

Intersectionality theory provides a critical framework for understanding how overlapping social identities can jointly shape individuals’ experiences. It was popularised as a theoretical approach in sociology, stemming from legal scholarship and Black feminist theory, and was used to describe the unique situation of Black women in the United States of America [14]. The theory posits that social positioning jointly interacts to shape experience, forming interconnected systems of advantage or disadvantage, rather than acting in isolation [14]. When used as a theoretical lens, the concept provides an overarching theory to describe social complexity. As an analytical framework, it involves the systemic application of intersectional principles to research methodology. Different analytical approaches have emerged within intersectionality research, including intercategorical methods that compare outcomes across established social groups, and intracategorical approaches, that focus on experiences within particular intersections of disadvantage [15]. The theory has since been applied across disciplines and extended to intersecting social identities, considering the interactions between multiple personal, geographic, and economic factors [14, 16].

In healthcare, intersectionality theory has predominantly been applied in qualitative studies on marginalisation, identity, and lived experiences [17]. In contrast, there exists a much smaller quantitative literature on the application of intersectionality across health and care settings. The slower uptake in quantitative research in this field and others has been driven primarily by uncertainty around the use of appropriate statistical methods [16, 18]. In particular, previous studies have often failed to apply intersectionality theory in a meaningful way, while standard regression methods have struggled to capture the effects of overlapping social identities [16]. This has often led to social identities being reduced to isolated variables, compounded by limited access to rich data on multiple dimensions of identity. Previous reviews of these studies have either evaluated the entire quantitative literature that has applied intersectionality theory [16] or focused on particular societal groups [19]. The degree to which intersectionality theory has been applied across both qualitative and quantitative studies within specific health settings, particularly primary care, remains unclear.

Primary care, in particular, represents a crucial context to analyse through an intersectional lens for two key reasons. First, primary care services in many countries are often the gatekeepers and first point of access for individuals within the broader health and care system. Gatekeeping in primary care has impacts on the outcomes and expenditure of other types of healthcare [20]. Second, access to quality healthcare is fundamentally associated with improved health outcomes at the population level, and also plays a critical role in reducing health inequalities across social groups [21]. Particularly in universal healthcare systems, such as in England, mounting primary care pressures from rising patient demand and persistent challenges in recruiting and retaining General Practitioners (GPs) serve to amplify existing disparities in the access to and quality of primary care services, especially for socioeconomically deprived and ethnic minority populations, who already experience reduced access to GPs [3, 6, 22].

Accurately identifying among which societal groups inequalities fall, by foregrounding how structural dynamics such as racism, sexism, and classism shape patient-provider interactions, health behaviours, and outcomes, is crucial for effectively addressing health and care inequalities [23]. To address this gap, we conduct this scoping review, chosen for its suitability in mapping diverse study designs, populations, and outcomes, with the aim of systematically cataloguing how intersectionality theory has been applied to primary care settings, and to identify areas for future research. To achieve this aim, we first scope the literature to identify studies that investigate how multiple social identities combine to shape experiences or outcomes in primary care. This captures a broad spectrum of literature: ranging from explicit applications where intersectionality actively drives the methodology, to implicit applications that reference the concept as a contextual lens for compounding disadvantages, despite using traditional, additive tools. Second, we extract key information from the included studies to provide a detailed map of how intersectionality theory has been applied in this setting.

## Methods

We conducted our review in accordance with the PRISM-ScR reporting guidelines for scoping reviews [24]. Using the methodology outlined in Arksey & O’Malley [25] and Levac et al. [26], our approach consisted of five stages: (1) specify the research question; (2) identify relevant literature; (3) select studies; (4) map out the data; (5) summarise, synthesise, and report the results.

The protocol for this scoping review was published [27] is registered on Open Science Framework (OSF) registries (https://osf.io/h9p83*).*

### Stage 1: Specifying the research question

We identified the research question by examining how intersectionality theory and methods have been applied to identify and quantify intersectionality in primary care. While we initially considered the broader inequalities literature, we deliberately narrowed our scope to studies that mention intersectionality-related terminology, ensuring that our review remains focused on applications of the theory, both as a conceptual lens and within the methodological framework.

### Stage 2: Identifying relevant studies

To identify relevant literature, we conducted a comprehensive electronic search across six databases: Ovid MEDLINE, Embase, Econlit, PsycArticles, Social Policy and Practice (via OvidSP), and Scopus. This was supplemented by searching grey literature repositories, via OpenGrey, to identify a broader range of relevant non-peer reviewed studies.

We used an inclusive Boolean-based search strategy, integrating terms relating to primary care and intersectionality. The search strategy followed a protocol previously peer-reviewed and published [27], which utilised the search methodology established by Gill et al. [28]. By utilising this approach, we employed setting-specific terminology, rather than professional titles, to capture a broad range of primary care staff, while maintaining a clear distinction from secondary care. The search terms were subsequently iteratively refined to ensure relevance, as there are many different interchangeable terms for primary care. Details of the search terms, databases, and rationale, are provided in Table 1.

**Table 1:** Database, rationale, and search terms

Database	Rationale	Search syntax
Embase (Ovid)	Includes biomedicine and life science journals	1. ((“primary care”) OR (“primary health*”) OR (“general practi*”) OR (GP) OR (“family practi*”) OR (“family physician*”) OR (“family medic*”) OR (“health practi*”)) [ti, ab, key] 2. (intersection*) [ti, ab, key] 3. 1 AND 2
Ovid MEDLINE	Includes a range of journals in the health policy and public health fields	^a^
Econlit (Ovid)	Includes a range of journals in the social sciences field	^a^
APA PsycArticles (Ovid)	Includes a range of psychology journals	^a^
Social Policy and Practice (Ovid)	Includes social care journals	^a^
Scopus	Multidisciplinary nature including medicine and social science fields	1. TITLE-ABS-KEY ((“primary care”) OR (“primary health*”) OR (“general practi*”) OR (GP) OR (“family practi*”) OR (“family physician*”) OR (“family medic*”) OR (“health practi*”)) 2. TITLE-ABS-KEY (intersection*) 3. 1 AND 2
OpenGrey	Includes grey literature	^a^

^a^ Same search syntax to that presented in the first row

The final search, performed by SA, was conducted on the 20th of June, 2023, with no date restrictions to maximise the scope of the captured literature. We present the detailed search strategy in Table 2.

**Table 2:** Search strategy

Components	Search Items	Ovid Results	Scopus Results
#1	((“primary care”) OR (“primary health*”) OR (“general practi*”) OR (GP)) [TITLE-ABS-KEY]	825,066	491,562
#2	((“family practi*”) OR (“family physician*”) OR (“family medic*”) OR (“health practi*”)) [TITLE-ABS-KEY]	174,537	150,706
#3	#1 OR #2	920,660	566,453
#4	(intersection*) [TITLE-ABS-KEY]	62,076	191,853
#5	#3 AND #4	899	713
		**Total**	**1612**

### Stage 3: Study selection

We developed inclusion criteria following an initial familiarisation with the literature, to ensure that the scope and diversity of studies was accurately reflected. Given the extensive literature on primary care inequalities, we focused on studies that engage with intersectionality, encompassing both explicit applications, where the theory directly shapes the research design and methodology, and implicit applications, which employ it as a conceptual lens to examine compounding disadvantages. We used the definition of intersectionality defined by Bauer et al. [16], as a theoretical framework examining how multiple social identities interact, such as race, gender, and socioeconomic status, to create unique experiences of both privilege and disadvantage, thereby influencing health outcomes. Our criteria targeted peer-reviewed empirical studies within primary care settings that applied an intersectional approach, including quantitative, qualitative, and mixed-methods designs. We excluded non-empirical literature such as theoretical papers and commentaries, as these do not provide practical empirical evidence relevant to the objectives of this scoping review.

These inclusion criteria consisted of four key components. First, to ensure alignment with our research question, all studies were required to be situated within a primary care context. Second, we targeted research that specifically considered intersections within the analysed sample or population, ensuring that the study moved beyond single-axis analysis to explore more than one social characteristic in combination. Third, to maintain a focus on empirical evidence, we included qualitative, quantitative, and mixed-methods designs, while excluding non-empirical works such as theoretical papers or commentaries. Finally, due to resource constraints, the review was limited to studies published in English.

Following the search, we managed and identified records in Zotero (v6), where duplicates were systematically removed by SA. We then exported records into Microsoft Excel for screening.

Titles and abstracts were independently screened by six reviewers (HB, PB, PS, SG, SU, GC) who assessed eligibility against the inclusion criteria. A 10% sample of titles and abstracts was cross-verified by two reviewers (PB and SU) to ensure consistency and reviewer calibration. The team convened regularly to discuss the studies and resolve any discrepancies through consensus.

Studies meeting the initial criteria subsequently underwent a full text review by HB and SA, with any disagreements resolved by SU. Following this, SA conducted a forward and backward citation search in August 2024 to identify any potentially overlooked literature.

### Stage 4: Data collection

The authorship team developed a standardised data extraction form that was applied to the final set of included studies. The form captured key details, such as study demographics, methodologies, outcomes assessed, and specific intersections considered (along with quantity). The data extraction form and the rationale for each extracted dimension is detailed in Table 3.

**Table 3:** Data extraction form

Dimensions	Details	Reason
General information	Authors, year, and country of study	To examine the geographical spread and development over time of the literature
Dataset and study design	Quantitative, Qualitative, or Mixed-methods, and sample size	Identify the type of study and the specific dataset
Data analysis method	e.g. Descriptive, regression	Identify the type of method within quantitative, qualitative, or mixed-method study design
Study participants	Patients or Healthcare professionals	To examine if intersectionality has been applied to a patient or workforce primary care perspective
Study aim	Stated aim of the study and if intersectionality is part of this (/ these) aims	Identify if intersectionality is a primary aim of the study
Outcome	Who these outcomes relate to (e.g. patients, workforce)	Identify what type of outcome intersectionality analysis has been applied to
Intersections considered and the number of intersections	Social groups analysed and number of distinct social identities analysed in combination	To understand the breadth and depth of the intersections analysed
Type of intersectionality	Intercategorical, Intracategorical, or Anti-categorical	A key categorisation of intersectionality types
Results	Main findings of the study	

Data extraction was primarily conducted by HB, while SA independently verified the accuracy and consistency of the extracted information. To ensure reliability, interpretative judgements, such as those regarding the application of intersectionality, were discussed during meetings of the entire authorship team. Any further discrepancies or inconsistencies were resolved through consensus between SA and SU to ensure reliability. Following this process, SA coded the data for analytical purposes.

### Stage 5: Data summary and synthesis of results

The extracted data was analysed by SA, using descriptive statistics for study characteristics, and thematic analysis for qualitative patterns. Results were tabulated and accompanied by a contextual narrative outlining key findings, research gaps, and, crucially, implications for policy and practice. Consistent with scoping review methodology, the focus was on mapping the existing literature and its practical application, rather than formally appraising the quality of the literature itself.

To synthesise the literature, we drew upon two key frameworks to inform our analysis: (i) Creswell’s methodological classifications [29], which are commonly applied distinctions in academic research, and (ii) McCall’s threefold intersectionality schema, comprising intercategorical, intracategorical, and anti-categorical approaches [15]. Creswell categorises research into three broad types: quantitative, which tests theories by examining relationships among variables; qualitative, which explores how individuals make meaning of their experiences; and mixed methods, which integrate both approaches [29]. McCall adds further nuance by distinguishing between inter-, intra-, and anti-categorical approaches to intersectionality. Utilising McCall’s taxonomy, intercategorical approaches were defined as those that use existing social categories to examine and compare inequalities across different groups, for example between Black men and White women. Conversely, intracategorical approaches explore marginalised groups within specific intersections often overlooked by traditional frameworks, such as focusing on the unique experiences of Black transgender women [15]. Finally, anti-categorical approaches critique the notion of fixed social categories with simplistic labelling by treating identity as a fluid entity, thereby exposing the power dynamics created by categorical boundaries [15].

We drew on these distinctions to guide our interpretation of the literature for four reasons. First, they acknowledge the broad range of methodological approaches in intersectionality literature, from qualitative to quantitative designs. Second, the emphasis on intercategorical versus intracategorical perspectives reflects a fundamental distinction embedded in the intersectionality literature on whether to compare categories (intercategorical), or to centre research around a particular groups experience (intracategorical). Third, these distinctions allow for a more nuanced understanding of potential gaps in the literature, given that researchers often pre-specify their design on the basis of data availability. Finally, recognising these different approaches enables insights from one approach to inform theoretical and methodological developments in another.

### Patient and public involvement

The research question and focus of this review drew on discussion with the University of Manchester PRIMER (Primary Care Research in Manchester Engagement Resource) Patient and Public Involvement (PPI) group, as part of a larger research project on the application of intersectionality theory to identify inequalities in primary care.

## Results

### Search and selection process

**Fig. 1:**
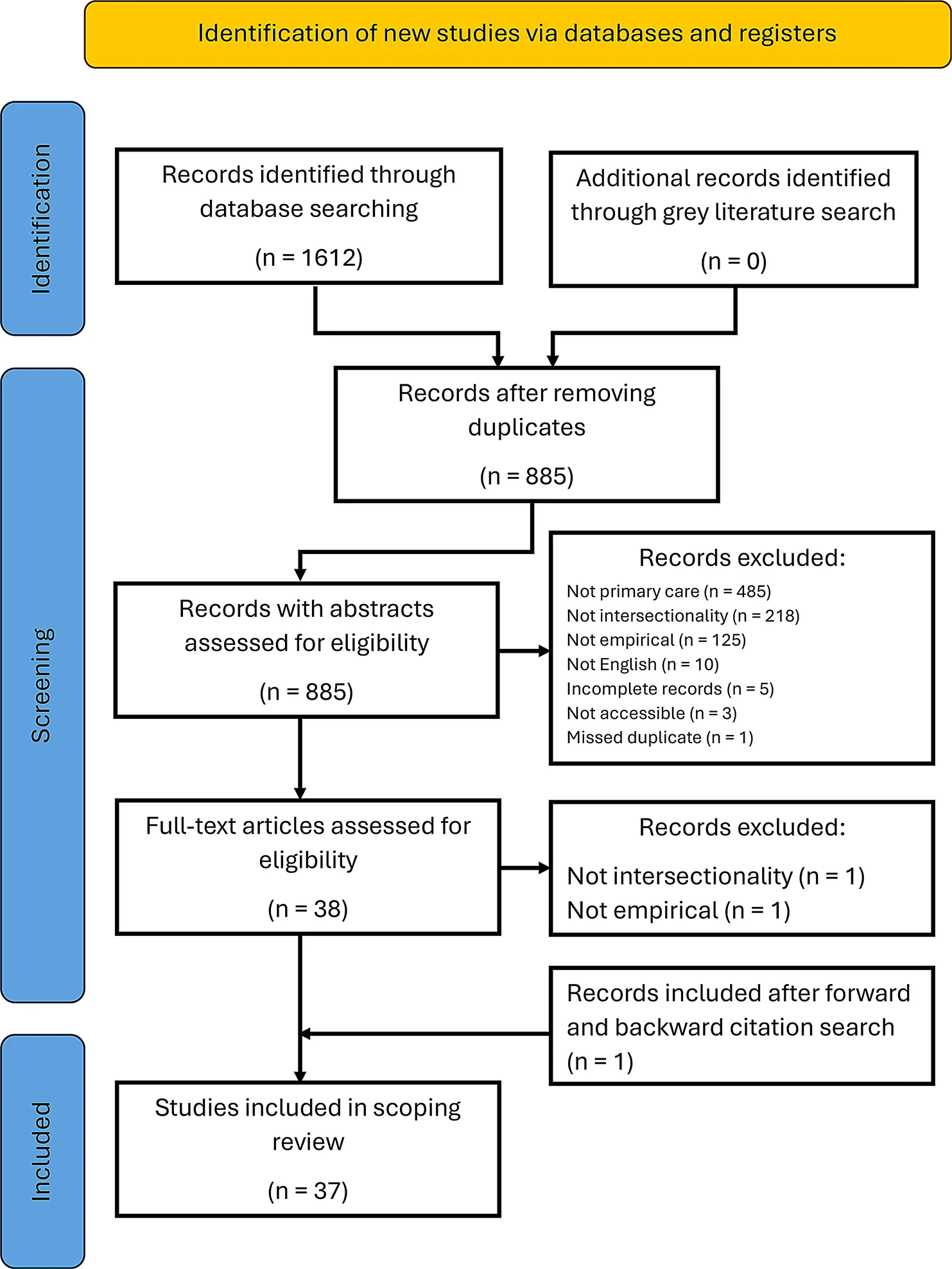
Study selection flow chart

We present the study selection process in Fig. 1. We identified 1612 records through database searches, with no additional studies retrieved from grey literature sources. After removing 727 duplicate records, 885 unique studies remained for initial screening. From these studies, we identified 38 studies as potentially meeting our predefined inclusion criteria after screening the titles and abstracts of each study. Following a full-text review, we classified 36 studies as meeting the inclusion requirements. We identified an additional study through forward and backward citation searching, resulting in a total of 37 studies.

### Study characteristics

We summarise the characteristics of the included studies in Table 4.

**Table 4:** Summary of study characteristics

Study characteristics (*N* = 37)		Count (%)
Year of Publication		
	2024	1 (2.7%)
	2023	4 (10.8%)
	2022	6 (16.2%)
	2021	6 (16.2%)
	2020	4 (10.8%)
	2019	4 (10.8%)
	2018	4 (10.8%)
	2017	3 (8.1%)
	2000–2016	5 (13.5%)
Country of study		
	United States of America	11 (29.7%)
	Canada	10 (27.0%)
	Australia	5 (13.5%)
	UK	4 (10.8%)
	Brazil	1 (2.7%)
	Denmark	1 (2.7%)
	Norway	1 (2.7%)
	Spain	1 (2.7%)
	Sweden	1 (2.7%)
	Uganda	1 (2.7%)
	Belgium / Italy / Malta / Portugal / Spain ^a^	1 (2.7%)
Size of dataset		
	< 100	15 (40.5%)
	100–999	6 (16.2%)
	1000–9999	6 (16.2%)
	10,000–99,999	4 (10.8%)
	100,000–999,999	5 (13.5%)
	>= 1,000,000	1 (2.7%)
Data type		
	Primary	23 (62.2%)
	Secondary	14 (37.8%)
Study design		
	Quantitative	20 (54.1%)
	Qualitative	15 (40.5%)
	Mixed-methods	2 (5.4%)
Data analysis method ^b^		
	Regression analysis	18 (48.6%)
	Descriptive statistics	5 (13.5%)
	Thematic analysis	10 (27.0%)
	Grounded theory	2 (5.4%)
	Content analysis	1 (2.7%)
	Framework analysis	1 (2.7%)
	Hermeneutic analysis	1 (2.7%)
	Interpretative phenomenological analysis	1 (2.7%)
	Unclear	1 (2.7%)
Study participants		
	Healthcare professionals	10 (27.0%)
	Patients	27 (73.0%)
Main aim of study		
	Intersectionality	29 (78.4%)
	Other	8 (21.6%)
Outcomes considered ^b^		
	Workforce related	4 (10.8%)
	Patient access	23 (62.2%)
	Patient use	8 (21.6%)
	Patient quality of care	14 (37.8%)
Intersections considered ^b^		
	Gender	27 (73.0%)
	Race / Ethnicity	23 (62.2%)
	Sexuality	12 (32.4%)
	Education	10 (27.0%)
	Socioeconomic status / Class	10 (27.0%)
	Immigration status	9 (24.3%)
	Age	5 (13.5%)
	Rurality	5 (13.5%)
	Religion	4 (10.8%)
	Housing status	4 (10.8%)
	Disability	3 (8.1%)
	Other	12 (32.4%)
Number of intersections		
	5	2 (5.4%)
	4	1 (2.7%)
	3	4 (10.8%)
	2	15 (40.5%)
	Unclear	13 (35.1%)
	Not stated	2 (5.4%)
Type of intersectionality considered		
	Intercategorical	25 (67.6%)
	Intracategorical	10 (27.0%)
	Inter- and intra-categorical	2 (5.4%)
	Anti-categorical	0 (0%)

^a^ combined study between multiple countries ^b^ several studies fit multiple categories

The 37 studies that met the inclusion criteria [30–66] were published between 2000 and 2024, with the majority published after 2017 (86.5%; *k* = 32) [30–32, 34, 36–42, 44–58, 61–66]. Geographically, research was concentrated in English-speaking countries including the United States [32, 34–35, 40–41, 44, 46, 48–49, 60, 63], and Canada (27%; *k* = 10) [30, 42–43, 45, 47, 50, 52, 61–63, 65], Australia accounted for 13.5% (*k =* 5) [31, 53, 55, 58, 64] and the United Kingdom (10.8%; *k =* 4) [33, 56–57, 59]. Sample sizes varied from six [56–57] to 5.3 million [45] participants with a large group of studies with less than < 100 participants (40.5%; *k =* 15) [49–63].The majority of studies (*k =* 23, 62.2%) [30–33, 49–65] utilised primary data, with secondary datasets comprising the remaining 37.8% (*k =* 14) [34–45, 48, 66].

Most studies focused on patients as the predominant participant group (73%; *k =* 27) [30–34, 36–45, 47–48, 54–62, 66], while healthcare professionals comprised the remaining 27% (*k =* 10) [35, 46, 49–53, 63–65]. Patient-related outcomes were central to the majority of studies, with a focus on access to primary care services, examined in 62.2% (*k =* 23) of cases [30–33, 37, 40–42, 46–48, 51, 53–59, 61–62, 65–66], service utilisation and barriers to accessing care were also common themes, in 21.6% (*k =* 8) of cases [34, 38–39, 41, 43–44, 47–48], alongside quality of care in 37.8% (*k =* 14) of studies [30, 34, 36–37, 45, 49, 52–53, 55–57, 59–60, 63]. Workforce-related outcomes, such as provider attitudes or experiences appeared in only 10.8% (*k =* 4) of the studies [35, 50, 64–65]. Intersectionality was the primary aim in most studies (78.4%; *k* = 29) [0–52, 54, 57–58, 61, 64–65]. The remaining studies (21.6%; *k =* 8) conceptually framed their research around intersectionality, but methodologically solely explored single social identities [53, 55–56, 59–60, 62–63, 66] rather than their intersections. These studies frequently acknowledged this discrepancy as a limitation, identifying the need for future research to transition away from single-identity exploration.

The 37 included studies explored the intersections of various social identities, most commonly gender (73%; *k =* 27) [30, 32, 35–36, 38–40, 42, 45, 47–55, 57–64, 66], race (62.2%; *k =* 23) [30–35, 40, 42, 44, 46–47, 49–53, 55–56, 59–61, 64–65], and sexuality (32.4%; *k =* 12) [30–31, 40–41, 50, 53, 55–58, 63, 65], which also formed the most common intersectional pairings. Most studies examined two intersections (40.5%; *k =* 15) [33–39, 41, 43–48, 60], fewer examined three (10.8%; *k =* 4) [32, 57–58, 62] or at least four (8.1%; *k =* 3) [31, 42, 66]. However, for a significant portion (40.5%; *k =* 15), the number of intersections considered was unclear or not explicitly stated [30, 40, 49–56, 59, 61, 63–65].

### Study characteristics by design and intersectionality approach

Study designs were most frequently quantitative (54.1%; *k =* 20) [30–48, 66], followed by qualitative (40.5%; *k =* 15) [49–62, 65], with only two studies utilising mixed-methods approaches [63–64]. Intercategorical frameworks dominated, which compare outcomes across predefined social groups (for example, race, gender, age, and immigration status [42]), in 67.6% (*k =* 25) of studies [30–45, 49–55, 63–64]. Meanwhile, intracategorical approaches, which focus on variation and nuance within a single social group (for example, within rural young people [55]), were applied in 27% (*k =* 10) of studies [46–48, 56–62], with the remaining 5.4% (*k =* 2) utilising both methods [65–66]. None of the studies included employed anti-categorical frameworks.

In this section we outline the characteristics of studies across their design (quantitative and qualitative) and intersectionality approach (intercategorical and intracategorical). We present studies across these categories in Table 5.

**Table 5:** Categorised approaches to intersectionality

*n* = 33 ^a^	Quantitative Intercategorical	Quantitative Intracategorical	Qualitative Intercategorical	Qualitative Intracategorical
Number of studies (proportion)	16 (43.2%)	3 (8.1%)	7 (18.9%)	7 (18.9%)
Data type	Primary (25%), Secondary (75%)	Primary (66.7%), Secondary (33.3%)	Primary (100%)	Primary (100%)
Sample size range	392–5.3 million	110–400	8–41	6–38
Data analysis method ^b^	Regression (87.5%), Descriptive (25%)	Regression (100%)	Thematic (71.4%), Grounded theory (14.3%), Hermeneutic (14.3%)	Thematic (57.1%), Grounded theory (14.3%), Content analysis (14.3%), IPA (14.3%)
Intersection-ality main aim	100% (*n* = 16)	100% (*n* = 3)	71.4% (*n* = 5)	42.9% (*n* = 3)
Commonly examined intersections ^b^	Race (56.3%), Gender (56.3%), Education (56.3%)	Race (66.7%), Gender (66.7%),	Race (100%), Gender (85.7%), Class (57.1%), Sexuality (42.9%)	Gender (85.7%), Race (57.1%), Sexuality (42.9%)
Number of intersections	2–5 2 (68.8%)	2 (100%)	Unknown (100%)	2–3 2 (14.3%), 3 (42.9%), Unknown (42.9%)
Outcomes assessed ^b^	Access (50%), Use (37.5%), Quality (31.3%), Workforce (6.3%)	Access (100%), Use (66.7%)	Access (57.1%), Use (57.1%), Workforce (14.3%)	Access (85.7%), Quality (57.1%)

^a^ excludes studies with both qualitative and quantitative study designs or utilising both intercategorical and intracategorical frameworks ^b^ several studies fit multiple categories

#### Quantitative intercategorical

Quantitative intercategorical studies, representing the largest category identified (43.2%; *k =* 16) [30–45], typically employed statistical methods on large, predominantly secondary (75%; *k* = 12), datasets [34–45]. Notably, the vast majority of research examining education of patients as an intersection (90%; *k* = 9 of 10) [30–33, 36–40] fell within this category, consistent with the standardised coding of this data as series of discrete, categorical variables.

#### Quantitative intracategorical

Quantitative intracategorical studies [46–48], though a smaller group, investigated healthcare experiences by specifically focusing on the complexities within specific, predefined intersections, rather than making broader comparisons between groups. These studies restricted their scope to the interaction between two axes of identity, predominantly to investigate healthcare access.

#### Qualitative intercategorical

Qualitative intercategorical studies [49–55] utilised their methods to explore and interpret the lived experiences and perspectives that contribute to health inequalities observed between different intersectional groups. In all of these studies, the number of intersections investigated was difficult to quantify, reflecting a tendency to view identity as a holistic, inseparable experience, rather than a sum of distinct demographic factors.

#### Qualitative intracategorical

Qualitative intracategorical studies [56–62] employed approaches to provide an understanding of the unique challenges and perspectives inherent to specific intersectional positions, rather than seeking to quantify differences across distinct groups. Notably, this category contained the fewest studies identifying intersectionality as a primary aim (42.9%; *k* = 3) [57–58, 61]. Instead, intersectional themes tended to emerge organically from the nuanced narratives of a single group, with gender (85.7%; *k* = 6) [57–62] serving as the predominant analytical lens.

### Comparisons across study design and intersectionality method

#### Comparisons across study design

We compared studies across design and methodology to reflect the diversity of the literature and the varied interpretations of intersectionality. This allowed us to identify whether specific research types are associated with more or less rigorous operationalisations of intersectionality, and to consequently identify specific gaps in the evidence base.

Quantitative studies primarily used secondary data (70%; *k* = 14 of 20) [34–45, 48, 66] and averaged 423,037 participants per study, while qualitative studies exclusively used primary data (100%; *k* = 15 of 15) [49–62, 65] from much smaller samples, averaging 43 participants. This methodological divide directly influenced the analytical approach. Quantitative studies focused on more historically recorded characteristics such as education level (50%; *k =* 10 of 20) [30–33, 36–40, 66] or immigration status (35%; *k =* 7 of 20) [33, 37, 41–43, 45, 66], whereas qualitative research delved into more fluid identities, like gender (86.7%; *k =* 13 of 15) [49–55, 57–62] and sexuality (46.7%; *k =* 7 of 15) [50, 53, 55–58, 65]. Quantitative studies predominantly examined two intersections (70%; *k =* 14 of 20) [33–39, 41, 43–48], while the number of intersections remained unclear or unstated for most qualitative studies (73.3%; *k =* 11 of 15) [49–56, 59, 61, 65]. Quantitative studies predominantly applied an intercategorical framework (80%; *k =* 16 of 20) [30–45], facilitating comparisons across predefined, intersecting social groups, a more amenable approach for quantitative data. In contrast, qualitative studies were more balanced in their approach between intercategorical (46.7%; *k =* 7) [49–55] and intracategorical (46.7%; *k =* 7) [56–62] frameworks.

This split was also reflected in the studies’ primary aims, with 95% (*k =* 19 of 20) of studies employing quantitative methods [30–48] explicitly focusing on intersectionality, but just 60% (*k =* 9 of 15) of qualitative studies [49–52, 54, 57–58, 61, 65]. Although patients were the predominant participant group in most studies, qualitative studies were more likely to include healthcare professionals (40%; *k =* 6 of 15 vs. 10%; *k =* 2 of 20). This focus was not reflected within the outcomes assessed, with workforce related outcomes receiving less focus in both study designs, making up just 13.3% (*k =* 2 of 15) of qualitative [50, 65], and 5% (*k =* 1 of 20) of quantitative studies [35]. Quantitative studies were also more likely to analyse differences in the quality of services (20% vs. 0%), possibly reflecting the availability of standardised performance metrics.

The two remaining studies utilised a mixture of both frameworks [63, 64]. The first study involved 63 United States-based participants, employing descriptive statistics and thematic analysis to investigate the preparedness of counsellors and psychologists to work with patients holding intersecting identities [63]. The second study involved 120 Australia-based participants, and examined service accessibility for ethnically diverse survivors of abuse [64]. Both utilised primary data, combining quantitative and qualitative approaches, although only the latter explicitly positioned intersectionality as its central focus.

#### Comparisons across intersectionality method

Intercategorical studies averaged 328,578 participants per study, while intracategorical studies averaged just 81, a disparity reflecting the intracategorical framework’s frequent application within qualitative research. Only intercategorical approaches considered workforce related outcomes (12%; *k =* 3 of 25) [35, 50, 64]. Both approaches focused heavily on patient access to services, but intercategorical approaches had a relatively greater emphasis on patient use (24%; *k =* 6 of 25) [34, 38–39, 41, 43–44] and quality (40%; *k =* 10 of 25) of services [30, 34, 36–37, 45, 49, 52–53, 55, 63], a similar pattern to that observed in quantitative studies.

Intersectionality was more likely to be a main aim in intercategorical (88%; *k =* 22 of 25) [30–45, 49–52, 54, 64] compared to intracategorical (60%; *k =* 6 of 10) studies [46–48, 57–58, 61]. Intracategorical studies almost exclusively focused on three main intersections - race (60%; *k =* 6 of 10) [46–47, 56, 59–61], gender (80%; *k =* 8 of 10) [47–48, 57–62], and sexuality (30%; *k =* 3 of 10) [56–58] - while those employing intercategorical frameworks showed a more varied distribution. Both frameworks primarily focused on 2 intersections, when data was available, but interestingly, two intercategorical studies (8%) examined 5 intersections each [31, 42]. The remaining two studies combined both intercategorical and intracategorical approaches [65, 66].

We document the individual characteristics of the 37 included studies in the *appendix*.

## Discussion

### Main findings

This scoping review mapped the application of intersectionality frameworks within primary care research, identifying 37 studies. Our findings highlighted a growing application of the theory, but the explicit application of intersectionality to inform the design and methodological approach in primary care studies still lags behind other fields [16]. Methodologically, this application was split between quantitative studies favouring intercategorical comparisons, and qualitative studies employing a more balanced mix of approaches. Notably, no studies used an anti-categorical framework. Race and gender dominated the examined intersections, often combined with socioeconomic status or sexuality, with research largely centred on patient outcomes such as access and care quality. Substantial gaps remain, particularly regarding provider perspectives and workforce outcomes, despite the importance of provider interactions in primary care. Geographically, the identified research was heavily concentrated in high-income English-speaking countries such as North America, the UK and Australia. Additionally, there was a lack of studies from low and middle-income or culturally diverse settings which currently under-represents some contexts, such as indigenous populations.

The operationalisation of intersectionality varied widely. Our interpretative observation found that in many studies, it functioned more as an overarching theory to explain studies results rather than an analytical tool. This distinction became particularly apparent during our review, with many authors invoking intersectionality only in the discussion to interpret findings, rather than embedding the theory’s core logic into their primary statistical or qualitative analyses. Furthermore, the empirical execution of the theory remained inconsistent: qualitative studies often drew on intersectionality without specifying discrete identity combinations, while quantitative studies largely relied on stratification or two-way interactions rather than other established methods [67]. In addition, 73.3% of studies contained unclear or unstated variables, namely intersections, creating significant ambiguity and obscuring important nuances. Ultimately, this heterogeneity limits our ability to interpret the depth of these analyses, and hinders comparability across studies, even as it underscores the broad, albeit varied, applicability of intersectionality in primary care research.

### Strengths and limitations

We provide the first comprehensive review of how intersectionality theory has been applied as a framework to evaluate healthcare inequalities in the primary care setting. Our review included different study designs (qualitative and quantitative) and implemented a broad search strategy across multiple databases and grey literature [24, 25]. By utilising the search methodology established by Gill et al. [28], we employed setting-specific terms rather than professional titles; this approach successfully captured a broad range of primary care staff, including nursing and allied health professionals, while maintaining a clear boundary from secondary care. The categorisation of studies by intercategorical or intracategorical approach and quantitative or qualitative design ensures future analysis on this area has focused evidence to draw on, given the diversity within the included studies. This enabled us to map the existing literature and identify key gaps and emerging trends in the application of intersectionality, grouped based on approach and methodology.

However, several limitations must be acknowledged. Most notably, requiring intersectionality-related terminology potentially excluded relevant studies incorporating its principles using different language or conceptualisations. Therefore, this review should not be read as encompassing all knowledge on multiple, compounding disadvantages in primary care, but rather focuses on studies framed as intersectional under the chosen terminology. Additionally, the English-language restriction likely biased the findings towards English speaking settings, contributing to the observed geographic distribution and potentially excluding studies that do not define “primary care” that fits with our search terms. Furthermore, we intentionally did not restrict our search by publication year; while this captured older studies with varying levels of theoretical depth, it provided a more comprehensive ‘scoping’ of the literature, allowing us to observe the historical evolution of the field and identify how the rigor and depth of intersectional applications have matured over time. As a scoping review, we did not formally appraise study quality or perform a meta-analysis given the heterogeneity in studies. Finally, categorising study approaches involved some simplification, which may have overlooked nuances in study type or methodology; however, the categories we used reflect common approaches to classifying the type of intersectionality.

### Implications of the results and future directions

Our review of intersectionality research within healthcare builds upon other reviews on the application of intersectionality. We confirm Bauer et al.’s central finding that intersectionality is often poorly operationalised [16]. Our interpretative observation found that in many studies, this manifested as a heavy reliance on simple stratification of two-way interaction models, with the term “intersectionality” often appearing only as a post-hoc lens in the discussion rather than driving the methodology. Furthermore, our findings show a persistent failure to engage with the theory’s foundational principles, such as those articulated by Crenshaw, who argues for a focus on systems of power [14]. Instead, we found that primary care studies rarely moved beyond individual patient characteristics to examine how structural drivers shape health outcomes. This analytical focus on individuals is even more apparent when viewed through Roth’s lens, which frames intersectionality as a tool for political action [68]. This perspective starkly highlights the absence of intervention research in our findings; specifically, we identified no Randomised Controlled Trials (RCTs) or system-wide policy evaluations, revealing a critical gap between merely describing intersectional disparities and empirically testing strategies to resolve them.

Building on this, we identified several specific limitations in the current body of literature. There is limited generalisability of the current studies by the intersections considered, geography of the studies and choice of outcomes. Comparative studies across different health systems could be especially useful in understanding how systemic features mediate intersectional inequalities. There was a lack of intervention research (such as randomised controlled trials). Studies have primarily described or explored inequalities, but few have designed, implemented, or evaluated interventions or services specifically targeting intersectional disparities. Furthermore, future research could broaden the framing of structural determinants beyond social identities to include contextual and environmental dimensions; integrating these physical and spatial factors into intersectional models would provide a more comprehensive understanding of health equity [69].

Given the range of methodologies the implications of our results should reflect this. For quantitative studies, most commonly applied methods such as stratifications or two-way interaction methods may not fully capture the multiplicative effects central to intersectionality theory. However, researchers must navigate a delicate trade-off of increasing the number of intersecting categories (for completeness) but simultaneously shrinking subgroup sizes which reduces statistical power [42]. We advocate for a principle of methodological alignment to navigate this complexity, which begins with a deep interrogation of the research aims, and definition of a core analytical goal. This requires a conscious choice: either limit intersections to ensure statistical robustness in smaller datasets or, when granular detail is essential, proactively leveraging large-scale datasets (such as surveys and electronic health records), along with new analytical approaches beyond simple interaction terms. This strategy prevents the masking effects of single-axis models, which can misrepresent vulnerability and obscure disparities within smaller subgroups [39]. As demonstrated by a recent EU study [66], intersectional re-analysis of existing datasets can uncover specific patterns of disadvantage that go unnoticed or under-appreciated when using conventional, additive methodologies.

Several promising methods have emerged to facilitate this more rigorous approach, including data-driven decision trees, chi-square automatic interaction detection models, and most recently multilevel regression models, like MAIHDA [16, 67]. Adopting any of these more rigorous methods is a critical step, as it allows for the formal testing of hypotheses about intersectional disadvantage, thereby providing the robust evidence needed to move from description to intervention. By leveraging existing datasets to identify populations facing compounded barriers, service delivery can be tailored, such as through extended appointments or increased resource allocation, specifically for those currently falling through the cracks of existing health metrics.

Qualitative research can continue to provide crucial depth and context, particularly exploring the mechanisms through which intersecting identities shape experiences and outcomes within primary care services. Integrating theoretical advancements that consider structural or political forms of intersectionality is key. For example, qualitative inquiry could explore how a policy like the UK’s Quality and Outcomes Framework (QOF), which incentivises specific dimensions of clinical quality, such as single-category health checks, might inadvertently fail patients at the intersection of multiple disadvantages. This approach would shift the focus from individual patient behaviours to institutional practice [68].

The absence of anti-categorical frameworks represented a significant methodological gap. While this approach is inherently suited to challenging fixed social categories, consequently exposing underlying power dynamics, its current omission from the literature likely reflects the inherent challenges of applying such a fluid concept within structured empirical analyses. Further methodological development is ultimately needed to create feasible ways of applying anti-categorical insights in both qualitative and quantitative research. Developing such methods is essential to facilitate progress beyond simply analysing individual-level factors to tackling the institutional power dynamics and systemic barriers that underpin health inequalities.

While intersectionality prompts consideration of structural factors, such as racism, sexism, and classism, that shape power dynamics and consequently impact patient-provider interactions, health behaviours, and health outcomes [59], it must be applied more rigorously to healthcare providers themselves. Research focusing on professionals is scarce, which is a significant gap because providers are not neutral observers, but active participants within the institutional systems that create inequities. In practice, this points to a need for clinical communication training that is intersectionally informed, moving beyond basic cultural competency to help providers identify how their own personality and unconscious biases shape the consultation. Future intersectional research focused on the healthcare workforce can move beyond attributing disparities solely to individual patient characteristics, instead highlighting how provider identity, bias, and clinical decision-making actively shape patient-provider interactions and contribute to systemic inequities [35, 50, 64, 65]. Such work is essential to transform intersectionality from a tool for diagnosing patient-level problems into one that informs structural interventions within the primary care system itself.

Adopting these intersectional methods provides primary care with a concrete framework to translate ‘hard-to-reach’ data into actionable service improvements. Practically, moving beyond descriptive analysis allows service managers to implement needs-led resource prioritisation, directing extended appointments and bespoke outreach specifically to those at the intersections of greatest disadvantage. This directly improves service impact by challenging the ‘one-size-fits-all’ logic of standard metrics, such as the Quality and Outcomes Framework (QOF), which often overlooks compounded barriers. Ultimately, these shifts move the responsibility for access away from the individual and into the core design of the system, ensuring that clinical capacity is deployed according to the compounded burden of inequality, rather than the narrow scope of current health indicators.

## Conclusion

In this scoping review we find that while the application of intersectionality in primary care research is still evolving, it already offers value for understanding and addressing health inequalities. An intersectional lens provides the potential for new insights into the complex nature of disadvantage that is frequently overlooked. For clinicians, it underscores the need for tailored communication and understanding about how intersecting identities shape patient experiences and potential biases. For primary care managers and public health practitioners, intersectionally analysed local data can guide more precise identification of underserved populations. For policymakers, it means enabling targeted resource allocation to better support populations facing complex needs. Moving beyond single-factor analyses with an intersectionality approach offers a crucial tool to assist primary care to fulfil its potential as a cornerstone of equitable health systems for all. By providing a clear map of the field and its methods, this review offers a foundation for developing the more rigorous, structurally-aware, and intervention-focused research needed to realise the full potential of intersectionality in primary care.

## Supplementary Information

Below is the link to the electronic supplementary material.

Supplementary Material 1: Appendix. Characteristics of included studies

## Data Availability

All data generated or analysed during this study are included in this published article (and its supplementary information files).

## Declarations

### Patient and public involvement

Patients and/or the public were involved in the design, or conduct, or reporting, or dissemination plans of this research. Refer to the Methods section for further details.

### Provenance and peer review

Not commissioned; externally peer reviewed.
