# Supplementary material for: A scoping review on the application of intersectionality as an analytical approach in primary care research
Source: BMC Health Serv Res. 2026 Aug 3;26:1507. doi: 10.1186/s12913-026-14873-9 (PMC13725955; doi:10.1186/s12913-026-14873-9)
Supplement: Supplementary file 1 [file 12913_2026_14873_MOESM1_ESM.docx]

| **Appendix 1: Characteristics of included studies** | | | | | | | | | | | | | |
| --- | --- | --- | --- | --- | --- | --- | --- | --- | --- | --- | --- | --- | --- |
| **Study no.** | **Author details** | **Year of publication** | **Country of study** | **Data type** | **Dataset** | **Study design** | **Data analysis method** | **Study participants** | **Did study main aim include intersectionality?** | **Study outcomes** | **Intersections considered** | **Number of intersections** | **Type of intersectionality** |
| 1 [30] | Gahagan, J.; Subirana-Malaret, M. | 2018 | Canada | Primary | 293 | Quantitative | Descriptive statistics | Patients | Yes | Patient access  Patients quality of care | Race  Gender  Sexuality  Education  Employment status | Not stated | Intercategorical |
| 2 [31] | Filia, K.; Menssink, J.; Gao, C.X.; Rickwood, D.; Hamilton, M.; Hetrick, S.E.; Parker, A.G.; Herrman, H.; Hickie, I.; Sharmin, S.; McGorry, P.D.; Cotton, S.M. | 2022 | Australia | Primary | 1107 | Quantitative | Regression analysis | Patients | Yes | Patient access | Race  Sexuality  Education  Rurality  Housing | 5 | Intercategorical |
| 3 [32] | Garcia M.E.; Hinton L.; Gregorich S.; Livaudais-Toman J.; Kaplan C.P.; Karliner L.S. | 2019 | USA | Primary | 1149 | Quantitative | Regression analysis | Patients | Yes | Patient access | Race  Gender  Education | 3 | Intercategorical |
| 4 [33] | Gazard, B.; Frissa, S.; Nellums, L.; Hotopf, M.; Hatch, S.L. | 2015 | UK | Primary | 1698 | Quantitative | Regression analysis | Patients | Yes | Patient access | Race  Immigration status  Education  SE status | 2 | Intercategorical |
| 5 [34] | Thorsen, M.; McGarvey, R.; Thorsen, A. | 2020 | USA | Secondary | 1229 | Quantitative | Regression analysis  Descriptive statistics | Patients | Yes | Patient use  Patients quality of care | Race | 2 | Intercategorical |
| 6 [35] | Corbie-Smith, G.; Frank, E.; Nickens, H. | 2000 | USA | Secondary | 4501 | Quantitative | Descriptive statistics | Healthcare professionals | Yes | Workforce | Race  Gender | 2 | Intercategorical |
| 7 [36] | Riiser S.; Haukenes I.; Baste V.; Smith-Sivertsen T.; Hetlevik O.; Ruths S. | 2021 | Norway | Secondary | 8857 | Quantitative | Regression analysis | Patients | Yes | Patients quality of care | Gender  Education | 2 | Intercategorical |
| 8 [37] | Peat, G.; Yu, D.; Grønne, D.T.; Marshall, M.; Skou, S.T.; Roos, E.M. | 2023 | Denmark | Secondary | 18,448 | Quantitative | Regression analysis | Patients | Yes | Patient access  Patients quality of care | Immigration status  Education | 2 | Intercategorical |
| 9 [38] | Cabezas-Rodríguez, A.; Bacigalupe, A.; Martín, U. | 2020 | Spain | Secondary | 20,313 | Quantitative | Regression analysis | Patients | Yes | Patient use | Gender  Education  SE status  Age | 2 | Intercategorical |
| 10 [39] | Nyamande, F.N.; Mosquera, P.A.; San Sebastián, M.; Gustafsson, P.E. | 2020 | Sweden | Secondary | 22,997 | Quantitative | Regression analysis | Patients | Yes | Patient use | Gender  Education | 2 | Intercategorical |
| 11 [40] | Kattari, S.K.; Call, J.; Holloway, B.T.; Kattari, L.; Seelman, K.L. | 2021 | USA | Secondary | 27,715 | Quantitative | Regression analysis | Patients | Yes | Patient access | Race  Religion  Gender  Sexuality  Disability  Education  SE status  Housing  Age  Military status  Overall health | Unclear | Intercategorical |
| 12 [41] | Gonzales, G.; Dedania, R.; Driscoll, R. | 2019 | USA | Secondary | 100,667 | Quantitative | Regression analysis | Patients | Yes | Patient access  Patient use | Immigration status  Sexuality | 2 | Intercategorical |
| 13 [42] | He, J.W.; Terry, A.L.; Lizotte, D.; Bauer, G.; Ryan, B.L. | 2024 | Canada | Secondary | 214,445 | Quantitative | Regression analysis  Descriptive statistics | Patients | Yes | Patient access | Race  Gender  Immigration status  SE status  Age | 5 | Intercategorical |
| 14 [43] | Durbin, A.; Moineddin, R.; Lin, E.; Steele, L.S.; Glazier, R.H. | 2015 | Canada | Secondary | 505,417 | Quantitative | Regression analysis | Patients | Yes | Patient use | Immigration status  SE status | 2 | Intercategorical |
| 15 [44] | Mares, J.G.; Lund, B.C.; Adamowicz, J.L.; Burgess, D.J.; Rothmiller, S.J.; Hadlandsmyth, K. | 2023 | USA | Secondary | 2,135,216 | Quantitative | Regression analysis | Patients | Yes | Patient use | Race  Rurality | 2 | Intercategorical |
| 16 [45] | Rijal H.; Kapral M.; Yu A.; Chu A.; Yu B.; Fang J.; Austin P.; Vyas M. | 2021 | Canada | Secondary | ~5.3 million | Quantitative | Regression analysis | Patients | Yes | Patients quality of care | Gender  Immigration status | 2 | Intercategorical |
| 17 [46] | Hull, S.J.; Tessema, H.; Thuku, J.; Scott, R.K. | 2021 | USA | Primary | 160 | Quantitative | Regression analysis | Healthcare professionals | Yes | Patient access | Race  Substance use | 2 | Intracategorical |
| 18 [47] | Scheim, A.I.; Zong, X.; Giblon, R.; Bauer, G.R. | 2017 | Canada | Primary | 400 | Quantitative | Regression analysis | Patients | Yes | Patient access  Patient use | Race  Gender | 2 | Intracategorical |
| 19 [48] | Goldenberg, T.; Jadwin-Cakmak, L.; Popoff, E.; Reisner, S.L.; Campbell, B.A.; Harper, G.W. | 2019 | USA | Secondary | 110 | Quantitative | Regression analysis | Patients | Yes | Patient access  Patient use | Gender | 2 | Intracategorical |
| 20 [49] | Lacombe-Duncan, A.; Berringer, K.R.; Green, J.; Jacobs, A.; Hamdi, A. | 2022 | USA | Primary | 8 | Qualitative | Thematic analysis | Healthcare professionals | Yes | Patients quality of care | Race  Gender  SE status | Unclear | Intercategorical |
| 21 [50] | Rezaiefar P.; Abou-Hamde Y.; Naz F.; Alborhamy Y.S.; LaDonna K.A. | 2022 | Canada | Primary | 10 | Qualitative | Thematic analysis | Healthcare professionals | Yes | Workforce | Race  Religion  Gender  Sexuality  Disability  SE status | Not stated | Intercategorical |
| 22 [51] | Camilo, C.; Kahhale, E.; Ferreira, M.L.; Schveitzer, M. | 2021 | Brazil | Primary | 11 | Qualitative | Hermeneutic analysis | Healthcare professionals | Yes | Patient access | Race  Gender  SE status | Unclear | Intercategorical |
| 23 [52] | Loncar, N.; Scott, K.L. | 2022 | Canada | Primary | 17 | Qualitative | Thematic analysis | Healthcare professionals | Yes | Patients quality of care | Race  Gender  SE status | Unclear | Intercategorical |
| 24 [53] | Robards, F.; Kang, M.; Tolley, K.; Hawke, C.; Sanci, L.; Usherwood, T. | 2018 | Australia | Primary | 22 | Qualitative | Grounded theory | Healthcare professionals | No | Patient access  Patients quality of care | Race  Gender  Immigration status  Sexuality  Rurality  Housing | Unclear | Intercategorical |
| 25 [54] | Ikhile, D.; Omodara, D.; Seymour-Smith, S.; Musoke, D.; Gibson, L. | 2022 | Uganda | Primary | 25 | Qualitative | Thematic analysis | Patients | Yes | Patient access | Religion  Gender  Social relations  Professionalism | Unclear | Intercategorical |
| 26 [55] | Robards, F.; Kang, M.; Steinbeck, K.; Hawke, C.; Jan, S.; Sanci, L.; Liew, Y.Y.; Kong, M.; Usherwood, T. | 2019 | Australia | Primary | 41 | Qualitative | Thematic analysis | Patients | No | Patient access  Patients quality of care | Race  Gender  Immigration status  Sexuality  Rurality  Housing | Unclear | Intercategorical |
| 27 [56] | Kelsall-Knight, L. | 2021 | UK | Primary | 6 | Qualitative | Thematic analysis | Patients | No | Patient access  Patients quality of care | Race  Sexuality  Adoptive parent | Unclear | Intracategorical |
| 28 [57] | Semlyen, J.; Ali, A.; Flowers, P. | 2018 | UK | Primary | 6 | Qualitative | Interpretative phenomenological analysis | Patients | Yes | Patient access  Patients quality of care | Religion  Gender  Sexuality | 3 | Intracategorical |
| 29 [58] | Lucas J.J.; Afrouz R.; Brown A.D.; Epstein S.; Ryan J.; Hayward J.; Brennan-Olsen S.L. | 2023 | Australia | Primary | 8 | Qualitative | Thematic analysis | Patients | Yes | Patient access | Gender  Sexuality | 3 | Intracategorical |
| 30 [59] | Sidhu, M.S.; Griffith, L.; Jolly, K.; Gill, P.; Marshall, T.; Gale, N.K. | 2016 | UK | Primary | 17 | Qualitative | Content analysis | Patients | No | Patient access  Patients quality of care | Race  Gender  Age | Unclear | Intracategorical |
| 31 [60] | Sacks, T.K. | 2013 | USA | Primary | 30 | Qualitative | Grounded theory | Patients | No | Patients quality of care | Race  Gender  SE status | 2 | Intracategorical |
| 32 [61] | Dahal, R.; Thapa Bajgain, K.; Bahadur Bajgain, B.; Adhikari, K.; Naeem, I.; Chowdhury, N.; Turin, T.C. | 2022 | Canada | Primary | 34 | Qualitative | Thematic analysis | Patients | Yes | Patient access | Race  Gender | Unclear | Intracategorical |
| 33 [62] | Clark, N. | 2018 | Canada | Primary | 38 | Qualitative | Thematic analysis | Patients | No | Patient access | Gender  Language  Health literacy | 3 | Intracategorical |
| 34 [63] | Dispenza, F.; Varney, M.; Golubovic, N. | 2017 | USA | Primary | 63 | Mixed-Methods | Descriptive statistics  Thematic analysis | Healthcare professionals | No | Patients quality of care | Gender  Sexuality | Unclear | Intercategorical |
| 35 [64] | Sawrikar, P. | 2020 | Australia | Primary | 120 | Mixed-Methods | Unclear | Healthcare professionals | Yes | Workforce | Race  Gender | Unclear | Intercategorical |
| 36 [65] | Roach, P.; Ruzycki, S.M.; Hernandez, S.; Carbert, A.; Holroyd-Leduc, J.; Ahmed, S.; Barnabe, C. | 2023 | Canada | Primary | 375 | Qualitative | Framework analysis | Healthcare professionals | Yes | Workforce  Patient access | Race  Sexuality  Rurality  Workplace  Leadership roles  Academic appointee | Unclear | Inter- and intra-categorical |
| 37 [66] | Rosano, A.; Dauvrin, M.; Buttigieg, S.C.; Ronda, E.; Tafforeau, J.; Dias, S. | 2017 | Belgium, Italy, Malta, Portugal, Spain | Secondary | 151,311 | Quantitative | Regression analysis | Patients | No | Patient access | Gender  Immigration status  Disability  Education | 4 | Inter- and intra-categorical |
